# Cross-cultural adaptation and psychometric properties of the Kessler Scale of Psychological Distress to a traumatic brain injury population in Swahili and the Tanzanian Setting

**DOI:** 10.1186/s12955-018-0973-0

**Published:** 2018-07-27

**Authors:** Joao Ricardo Nickenig Vissoci, Silvia Daniela Vaca, Deena El-Gabri, Leonardo Pestillo de Oliveira, Mark Mvungi, Blandina Theophil Mmbaga, Michael Haglund, Catherine Staton

**Affiliations:** 1 Duke Emergency Medicine, Duke University Medical Center, 8 Duke University Medical Center Greenspace, Durham, NC 27703 USA; 2 Department of Neurosurgery, Stanford University, Palo Alto, California USA; 3 Duke Global Health Institute, Duke University, Durham, North Carolina USA; 4 UniCesumar, Maringá, Paraná Brazil; 5 Kilimanjaro Christian Medical Center, Moshi, Tanzania; 6 Kilimanjaro Clinical Research Institute, Moshi, Tanzania; 7 Department of Neurosurgery, Duke University, Durham, NC USA

**Keywords:** Anxiety, Scale Validation, Psychometrics, Stress

## Abstract

**Background:**

To evaluate the psychometric properties of a Swahili version of the Kessler Psychological Distress scale in an injury population in Tanzania.

**Methods:**

Swahili version of the Kessler Psychological Distress scale was developed by translation and back-translation by a panel of native speakers of both English and Swahili. The translated instruments were administered to a sample of Tanzanian adults from a traumatic brain injury registry. The content validity, construct validity, reliability, internal structure, and external reliability were analyzed using standard statistical methods.

**Results:**

Both translated versions of the Kessler Psychological Distress scale were found to be reliable (>0.85) for all tested versions. Confirmatory factor analysis of one and two factor solution showed adequate results. Kessler Psychological Distress scale scores were strongly correlated to depression and quality of life (R>0.50).

**Conclusions:**

This paper presents the first Swahili adaptations of the Kessler Psychological Distress scale as well as the first validation of these questionnaires in Tanzania. The instrument was found to have acceptable psychometric properties, resulting in a new useful tool for medical and social research in this setting.

## Background

Psychological distress refers to the general concept of maladaptive psychological functioning in the face of stressful life events [1]. The defining attributes of psychological distress include perceived inability to cope effectively, change in emotional status, discomfort, communication of discomfort, and harm [2]. Therefore, psychological distress encompasses a variety of mental health disorders, most notably depression and anxiety. The lifetime prevalence of developing any common mental health disorder has been estimated to be 32.2% in high income countries and 25% in low and middle income countries worldwide, with the worldwide lifetime prevalence of anxiety disorders estimated to be 12.9% and that of depression to be between 10% and 15% [3, 4]. Depression is associated with a 20-fold mortality risk as compared to the general population [4]. Traumatic brain injury (TBI) patients have been shown to be susceptible to depressive symptoms and psychological distress. Studies have described higher correlations with anxiety and depression in patients with mild TBI compared to orthopedic trauma patients, as well as higher susceptibility to psychological distress regardless of injury severity [5–7]. Given the increases in morbidity and mortality associated with these disorders, identifying psychological distress in traumatic brain injury patients is of great importance.

Thus, an adequate evaluation of psychological distress is currently needed for the traumatic brain injury population. In Tanzania especially, no instrument to measure psychological distress has ever been validated and therefore there is a pressing need for a reliable objective measure to inform practice and policy. Also, further research is needed to provide more evidences of internal structure validity for the Kessler scale in traumatic brain injury patients.

One of the commonly and widely used instruments to measure psychological distress is the Kessler Psychological Distress Scale, which was originally developed in the United States for use in the National Health Interview Survey [8]. There are two versions of the original Kessler Scale - K10 and K6. The K10 contains ten questions asking how frequently the participant has experienced symptoms of psychological distress in the last 30 days, with the K6 consisting of a subset of six of these questions [9]. The Kessler scale has since been cross-validated to Asian and Pacific (Australia, New Zealand, Japan, Hong Kong, China, India), European (Netherlands, France, Italy, Romania, Bulgaria, Ukraine, Lebanon), North and South American (Mexico, Colombia, Brazil), and African (Nigeria, Burkina Faso, South Africa, Ethiopia) countries [10–24]. The initial validations and subsequent cross-validations have assessed the internal consistency and external validity of the Kessler Scale, confirming its acceptable psychometric properties. The Kessler scale has been shown to have an AUC ranging from 0.854 - 0.90 for the K10 and 0.865 - 0.89 for the K6 for any DSM IV disorders aside from substance use disorders [8, 25]. Factor analyses have supported both one and two dimensions, with the two dimensions characterized being anxiety and depression [11, 16, 26, 27]. Moreover, the Kessler Scale has been externally validated to several scales including the World Health Organization Disability Assessment Scale (WHODAS) [11], Center for Epidemiologic Studies Depression Scale (CES-D) [13], Mini International Neuropsychiatric Interview (MINI) [20], Hospital Anxiety and Depression Scale (HADS), and Hamilton Depression Rating Scale (HDRS) [26].

Nevertheless, few studies have evaluated the Kessler Scale’s validity with cross-cultural studies in Eastern Africa, or within the traumatic brain injury population. Arnaud has demonstrated the validity of the Kessler scale as a measure of patient-reported outcome for alcohol-related disorders in the emergency department setting [26], but a paucity of literature remains for TBI populations. Given the prevalence of the Kessler scale in assessments of psychological distress and the strong correlations between TBI and psychological distress, a reliable and validated version in Swahili would improve the ability to conduct quality studies in Tanzania, specifically with traumatic brain injury patients.

### Objective and Hypothesis

Thus, our aim was to report the psychometric properties of the first translation and adaptation of the Kessler Psychological Distress Scale to Swahili, specifically looking at (a) translating and performing content validity, (b) verify internal structure and consistency and (c) providing evidences of temporal and external validity.

## Methods

### Participants

Our sample was composed of 192 adults Traumatic Brain Injury patients from Moshi, Tanzania. Sample size was determined based on the recommendations of at least 5 to 15 subjects per item of the instrument [28]. Participants had an average age of 33.87 ± 13.32 years old and were mostly male (82.8%). Most patients (91%) showed mild injury severity, measured by GCS. Full demographic characteristics of study participants are presented in Table 1.

**Table 1:** Sociodemographic profile of the validation sample

VARIABLES	
Age (years), Mean (SD)	33.87 (13.32)
Household size, Mean (SD)	4.43 (2.48)
Monthly personal income, USD, Mean (SD)	$104.42 (100.08)
Monthly family income, USD, Mean (SD)	$155.20 (235.52)
Male, N (%)	159 (82.8)
Married, N (%)	104 (54.7)
Occupation, N (%)	
Business	44 (21.7)
Farming	41 (22.3)
Skilled worker	23 (12.5)
Salaried worker	67 (36.4)
Other	13 (7.1)
Education, N (%)	
Some primary education	112 (59.3)
Some secondary education	44 (23.3)
Some university education	33 (17.5)
TBI Severity (GCS), N (%)	
Mild	120 (90.9)
Moderate or Severe	12 (9.1)

### Instrument

The original scale used was the Kessler Psychological Distress Scale [9] which assesses Psychological Distress and consists of 10 unidimensional items (K-10). The answers are given on a 5-point Likert type scale (0 = None of the time to 4 = All of the time). The score is calculated by the sum of each item scores, ranging from 0 to 40 [8, 9]. Higher values ​​in each dimension mean high risk of psychological distress (anxiety or depression). The original scale was conceptualized to be unidimensional, however reports [16, 26, 27] found evidence of multidimensionality, specifically of two separated domains for anxiety and depression. A shorter version of the Kessler Psychological Distress Scale (K-6) was also developed, comprising 6 of the 10 items from the K-10 version [9].

### Ethical statement

The study was approved by the Institutional Review Board of the Duke University (IRB #Pro000061652), the Ethics Committee of the Kilimanjaro Christian Medical Center, Moshi, Tanzania and the National Institute for Medical Research (NIMR), Tanzania.

### Procedures

A translation and cross-cultural adaptation committee was formed by 5 judges (physicians, nurses and researchers); who freely participated to develop the translation, adaptation, and content validation process. After finalizing content validation, a pilot study was conducted with a group of 20 Tanzanian adults, selected by convenience, to assess the quality of instrument questions and coherence of language and content.

The instrument was translated through independent back translation methods. Initially, a native Swahili translator was hired to translate the Kessler questionnaires into Swahili. Subsequently, another bilingual translator was hired to convert the Swahili translated version back to English. English translated versions were compared with the original version of the instrument and checked for inconsistencies by 4 independent bilingual research nurses.

Issues with semantics were discussed and adjustments were made by the researchers and the judges committee. The experts’ opinions were initially collected individually and later discussed collectively to improve the quality of the translations and discuss any discordances.

### Data collection

Patients in the Kilimanjaro Christian Medical Center traumatic brain injury registry who were nearing their day of discharge from the hospital were screened for inclusion in this project. They were offered enrollment after informed consent in the local Swahili language, and were enrolled prior to discharge from the hospital. After informed consent, the patient had the Kessler Psychological Distress Scale questions administered at the patient bedside as a part of a 45 minute interview, establishing a baseline for future follow up evaluations. The interview consisted of a battery of tests involving mental health, substance use and cognitive functioning designed to validate and evaluate the constructs in Swahili and in the Tanzanian culture. Interviews were conducted at patient bedside prior to discharge, by a trained research nurse. In some instances, the interview happened over more than one sitting as delineated by patients’ status, stamina, and other needs related to their medical conditions. All responses were collected by hand and were entered into an internet based dataset (REDCAPS); all data entry had quality control performed by the principal investigator (CAS).

### Data analysis

Sociodemographic data were presented as means and standard deviations, medians, interquartile range, or absolute and relative frequencies. All analyses were conducted with R Language for Statistical Computing (R foundation, Vienna).

#### Reliability

Reliability is the capacity of an instrument to produce reliable results in different situations. We measured reliability with internal consistency to assess the degree to which all of the items in the instrument refer to the same subject [29] and temporal stability to assess the instrument's variation in time. To measure internal consistency we used a set of scores (Cronbach’s alpha, Omega and Composite Reliability), with coefficients above 0.7 considered acceptable [29].

#### Dimensionality and latent model

Parallel analysis was conducted to verify the literature pre-defined unidimensionality. Confirmatory factor analysis (CFA) was used to verify the construct validity of the instrument through: a) item-factor parameter and item-items individual reliability, b) absolute, incremental and parsimonious fit indexes; and c) average variance extracted to examine the convergent validity [29, 30].

CFA model adequacy was tested using Weighted Least Square Means and Variance Adjusted (WLSMV). Model adjustment was tested through the fit indexes (reference of expected values ​​for each index): Chi-square (X^2^ and p-value), Root Mean Square Error of Approximation (RMSEA <0.08, I.C. 90%), Tucker-Lewis index (TLI> 0.90), and Comparative Fit Index (CFI> 0.95). These indices aim to assess whether the model shows a good fit to the data, as proposed in the literature [30, 31]. Using a CFA approach, we tested two models for the K-10 (1, 2 and 3 factor solutions) and one model for the K-6 (Table 2). Convergent validity was assessed through the Average Variance Extracted (AVE) and values higher or close to .50 were considered acceptable indicators of convergent validity [32]. Discriminant validity was assessed by comparing the AVE with the squared correlation between factors. Composite reliability (CR) was calculated using CFA results, given that this measure provides the index of internal consistency of the instrument dimensions through the factor loadings of the respective items. Values greater than .70 were considered indicators of suitable composite reliability [33].

**Table 2:** Reliability (alpha), network centrality, factor adjustment (factor loading and thresholds) and difficulty parameter of Kessler's items

		K10	K6
	Threshold	Alpha	Alpha
*Depression*			
Q1. ... about how often did you feel tired out for no good reason?	1.82	0.89	
Q4. ... about how often did you feel hopeless?*	2.03	0.89	0.78
Q7. ... about how often did you feel depressed?	2.18	0.88	
Q8. ... about how often did you feel that everything was an effort?*	1.64	0.90	0.81
Q9. ... about how often did you feel so sad that nothing could cheer you up?*	1.93	0.89	0.77
Q10. ... about how often did you feel worthless?*	2.22	0.89	0.77
*Anxiety*			
Q2. ... about how often did you feel nervous?*	1.92	0.88	0.76
Q3. ... about how often did you feel so nervous that nothing could calm you down?	2.12	0.87	
Q5. ... about how often did you feel restless or fidgety?*	2.18	0.88	0.77
Q6. ... about how often did you feel so restless you could not sit still?	2.12	0.89	
Overall		0.88(0.86;0.90)	0.78(0.74;0.82)

*EC* Eigen centrality

* Questions inclued in the 6-item version of the instrument

#### External validity

Concurrent validity was assessed by the Spearman correlation between the Kessler Psychological Distress Scale measure and a set of mental health indicators (Quality of Life, Depression and Substance use). Quality of life was tested with the SF-8 Health Outcomes Scale [34] and has been previously shown to have a negative association with psychological distress in the literature. Depression was measured with the Patient Health Questionnaire (PHQ-9) [35] and CES-D [36] scales and we hypothesized a positive association with the Kessler Psychological Distress Scale in the literature [16, 26, 27].

## Results

Most of the participants were male (83%), married (55%), and had some primary education (59%). The average age (SD) was 33.87 (13.32) years old, with household sizes of 4.43 (2.48) people on average. Average personal monthly income among participants was 104.42 USD, which is slightly below the national living wage for an individual of 116.39 USD.

Internal consistency was adequate with scoring values above 0.85 for all parameters (Table 2) in both the K-10 and K-6 versions. Parallel analysis suggested 2 factors to be retained for the K-10 and confirmed the unidimensionality for the K-6. Items 10 (how often did you feel worthless**),** 7 (how often did you feel depressed) and 5 (how often did you feel restless or fidgety) had the higher threshold in identifying the latent trait, while item 8 (how often did you feel that everything was an effort**)** had the smaller threshold.

**Fig. 1:**
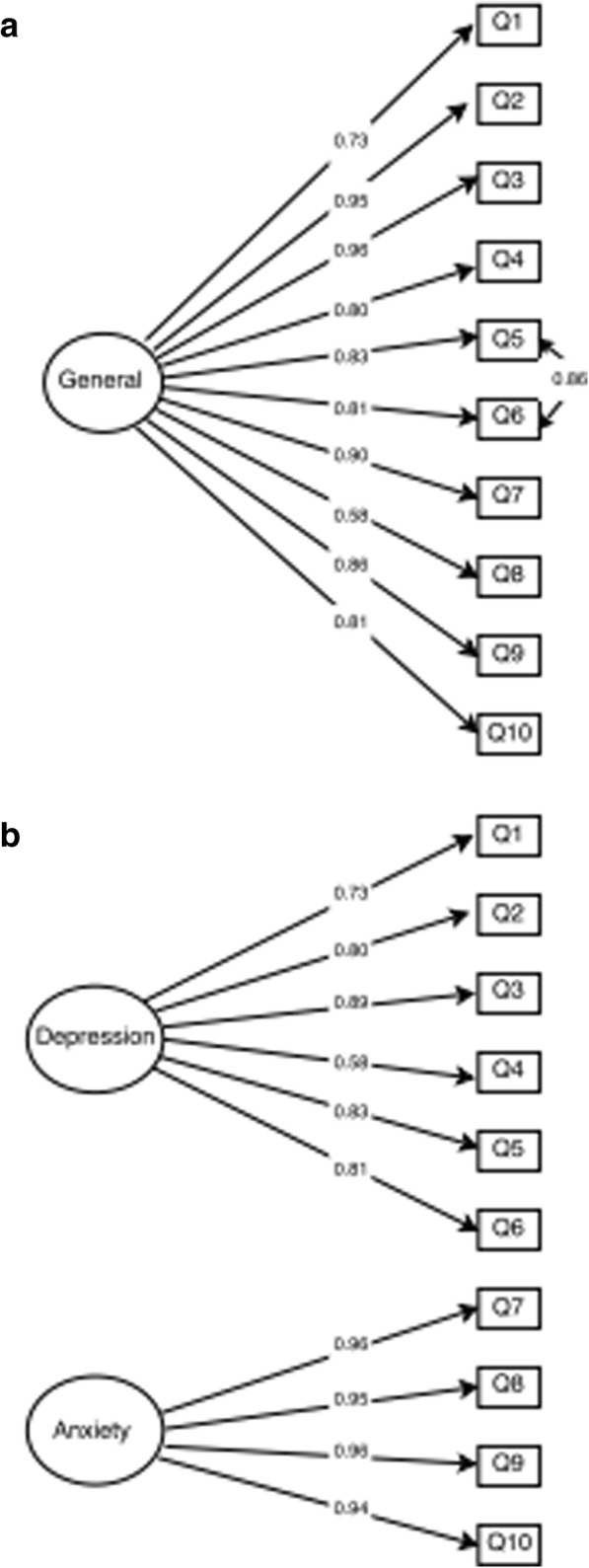
Internal structure of the Kessler Psychological Distress Scale: **a** Unidimensional K-10, and (**b**) two dimensions K-10.

**Fig. 2:**
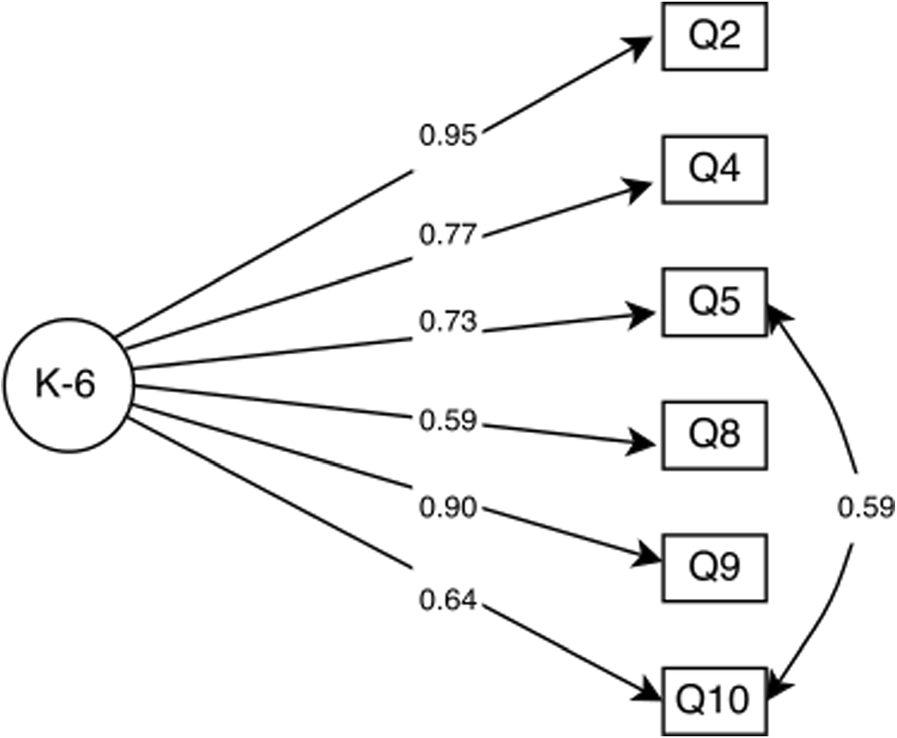
Internal structure of the Kessler Psychological Distress Scale K-6 items.

Confirmatory factor analysis showed that both one and two factor models had adequate fitness indicators (Table 3), showing acceptable structure in both options. Factor loadings indicate that individual item reliability was adequate for all items, ranging from 0.58 to 0.96 for the one factor model (Fig. 1a). The two factor model showed two independent constructs for Depression (FL ranging from 0.58 to 0.89) and Anxiety (FL ranging from 0.94 to 0.96) (Fig. 1b). The factor structure of the K-6 (Fig. 1a and b) showed adequate item reliability to the model (Fig. 2). Average variance extracted and Composite reliability also showed adequate values (Table 3).

**Table 3:** Psychometric properties results for construct validity

	X^2^ (Df)	RMSEA (95%)	TLI	CFI	AVE	CR
K-10						
1 Factor model	96.97 (35)	0.06 (0.04;0.08)	0.99	0.99	0.72	0.96
2 Factor model	90.27 (34)	0.07 (0.06;0.09)	0.98	0.98	0.63/0.91^a^	0.82/0.97^a^
K-6						
1 Factor model	11.60 (8) /0.170	0.04 (0.00;0.08)	0.99	0.99	0.61	0.90

*CFA* Confirmatory Factor Analysis, *X*^*2*^ Chi-Square, *Df* Degree of Freedom, *RMSEA* Root Mean Square Error of Approximation, *TLI* Tucker-Lewis Index, *CFI* Comparative Fit Index, *AVE* Average Variance Extracted, *CR* Composite Reliability

^a^Values for the Depression and Anxiety dimensions, respectively.

Multi-group Invariance for age, gender and education found that the constrained model with fixed factor weights in both groups showed similar structures adjustment in relation to the model with free parameters; suggesting that the model did not differ by group in relation to factor weights. The constrained model with fixed factor weights and covariance did not show significant change in comparison to the model with free parameters, suggesting no differences in the test's structure. Finally, the model with fixed coefficients differed significantly from the model with free structural coefficients when considering that the measurement model was invariant.

All Kessler Psychological Distress Scale scores showed a strong positive correlation with quality of life and negative with depression, performing as expected (Table 4).

**Table 4:** Correlation between K-10 and K-6 factor scores and depression and quality of life

	K-10*			K-6*
	Overall	Depression	Anxiety	
	R	R	R	R
Quality of life				
MCS	-0.53	-0.54	-0.51	-0.47
PCS	-0.54	-0.55	-0.52	-0.48
Depression				
CES-D	0.53	0.53	0.52	0.53
PHQ-9	0.59	0.61	0.58	0.54

*All correlations significant for a P<0.001

## Discussion

This is the first study to conduct a cross-cultural validation of the Kessler scale to Swahili. Besides, this is also the first study to show evidences of the psychometric properties of this scale in a traumatic brain injury sample. Most psychometric properties reports have been conducted with a general population or in perinatal women [19, 21, 23, 24]. In a general sense, the Swahili version of the Kessler Scale showed satisfactory psychometric properties, with adequate content, construct and external validity. Our results corroborated the results already available in the literature, thus preliminarily verifying a cross-cultural adaptation and validation of the Kessler Scale.

The Kessler Scale translated and adapted version showed values ​​of reliability similar to the ones found in the literature [11, 15, 16, 23, 24, 26]. Dimensionality measurement and factor analysis model reinforced the unidimensionality of the K-6 and suggested two dimensions for the K-10: anxiety and depression, though both one and two factor models had adequate fitness indicators. The original scale was reported to have unidimensionality (Kessler, 2002), yet several cross-validations have also supported the same dimensions (anxiety and depression) we observed [16, 27].

This paper also creates evidences not yet reported such as additional measures of internal consistency (composite reliability) and concurrent validity (SF-8 Health Outcomes Scale and PHQ-9). While Cronbach’s alpha is the most commonly used measure of internal consistency, it has been regarded as a lower bound estimate, underestimating true reliability if the measure is not tau-equivalent [37]. Composite reliability estimates reliability with structural equation modeling, thereby overcoming this limitation [38]. Reliability scores for each factor met internal consistency criteria present in the literature, being equal or higher than 0.70 [32, 39] and were also consistent to previous literature [11, 15, 16, 23, 24, 26].

Concurrent validity has been demonstrated between the Kessler Scale and the HADS, HDRS, CES-D, MINI, and WHODAS II (Arnaud, 2010; Sakurai, 2011; Spies, 2009, Validity of the K-10 in detecting DSM-IV-defined depression and anxiety disorders among HIV-infected individuals; Fassaert, 2009). Our results additionally show concurrent validity between the Kessler Scale and the SF-8 Health Outcomes Scale and the PHQ-9, expanding the number of mental health indicators correlated with the Kessler Scale.

One limitation with this study is related to its sample, which was composed of mostly male traumatic brain injury patients at the end of their hospital stay. This sample provides a limited perspective within the general population in Tanzania and thus the results presented must be considered within this scope. Future studies should approach these issues concerning cross validation in a way the results may be generalized to other independent samples in Tanzania and the general population. However, we chose the traumatic brain injury population specifically in Tanzania due to this population being underserved, under researched and with a growing population and need.

## Conclusions

Our results demonstrate that the Kessler Scale might provide relevant information to help healthcare providers in comprehending psychological distress amongst traumatic brain injury patients, allowing practitioners to understand different dimensions and potentially giving parameters to follow-up care of trauma patients in a psychological context. However, further studies should replicate the Kessler Scale psychometric properties testing to other samples and other cultures to confirm the factor solution stability found in our results, specifically with confirmatory approaches. Also, other psychometric properties need to be addressed such as measurement invariance, external validity, responsiveness or even individual item parameters.

Similarly, criterion validity should also be further tested to explore the quality of the scale as a predicting variable to depression and anxiety variables. Since this is the first instrument to be validated to Swahili, more research is needed to establish strong evidences of Kessler Scale behavior in relation to how psychological distress on this scale correlates with other psychological distress measures. Globally, the scale has shown good psychometric properties and could be used to assess psychological distress in the Tanzanian culture. This is an initial report on the psychometric properties of the Kessler Scale. It should lead to further development of a capacity to measure, and intervene, regarding psychological distress in Tanzania; allowing for more evidence-based practices and advance the research and policy development in this scenario.

## Abbreviations

AVE: average variance extracted
CES-D: Center for Epidemiologic Studies Depression Scale
CFA: confirmatory factor analysis
CR: composite reliability
HADS: Hospital Anxiety and Depression Scale
HDRS: Hamilton Depression Rating Scale
MINI: Mini International Neuropsychiatric Interview
PHQ-9: Patient Health Questionnaire
SD: standard deviation
TBI: traumatic brain injury
USD: US dollars
WHODAS II: World Health Organization Disability Assessment Scale
